# Supplementary material for: Synthetic Biology Toolkit for a New Species of Pseudomonas Promissory for Electricity Generation in Microbial Fuel Cells
Source: Microorganisms. 2023 Aug 9;11(8):2044. doi: 10.3390/microorganisms11082044 (PMC10458277; doi:10.3390/microorganisms11082044)
Supplement: Supplementary file 1 [file microorganisms-11-02044-s001.zip › microorganisms-2498381-supplementary.pdf]

Synthetic biology toolkit for a new species of *Pseudomonas* promissory for electricity generation in microbial fuel cells

Franciene Rabiço[1#], Matheus Pedrino[1#], Julia Pereira Narcizo[2], Adalgisa Rodrigues de Andrade[2], Valeria Reginatto[2] and María-Eugenia Guazzaroni[1*]

[1]Department of Biology, Faculty of Philosophy, Sciences and Letters of Ribeirão Preto, University of São Paulo, Ribeirão Preto, SP, Brazil

[2]Department of Chemistry, Faculty of Philosophy, Sciences and Letters of Ribeirão Preto, University of São Paulo, Ribeirão Preto, Brazil

*Correspondence to: María-Eugenia Guazzaroni, meguazzaroni@ffclrp.usp.br

[#]These authors contributed equally to this work.

Supporting Information

Figure S1. Segregational and structural stability of the tested plasmids as a function of replication origin in *Pseudomonas* sp. BJa5 strain.

Figure S2. Activity of the canonical P_{j100}, P_{j106} and P_{j114} promoter at 30°C.

Table S1. Transformation efficiencies of plasmids in the strain BJa5.

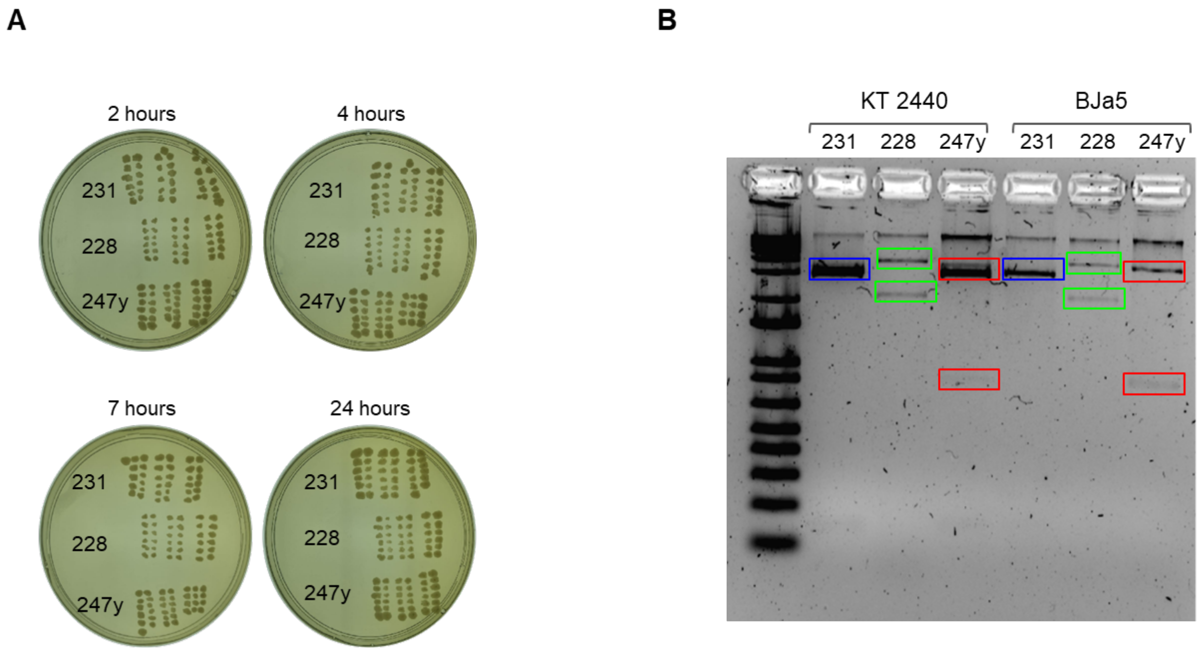

Figure S1. Segregational and structural stability of the tested plasmids as a function of replication origin in *Pseudomonas sp.* BJa5 strain. The strains *P. putida* KT2440 and *Pseudomonas sp.* BJa5 were grown in LB liquid medium without antibiotics for 2, 4, 7 and 24 h at 30 °C. After each of the periods, a solution of the diluted culture to 10^{-6} was plated on LB to obtain a countable number of CFU. Twelve colonies from each strain with the different plasmids were selected in each period, and their growth was evaluated on LB and LBKm plates. In Figure S1 A, it can be seen that all selected colonies of BJa5 grew on the LBKm plates. The assay was performed three times independently with equal results. In the Figure S1 B, the colonies from the 24 h period, had the plasmid DNA extracted and then digested with PacI and SpeI enzymes. The pattern obtained was analyzed by electrophoresis on agarose gels. The restriction patterns of BJa5 were compared with those obtained from plasmid from *P. putida* KT2440 and were found to be similar. This analysis was repeated twice independently.

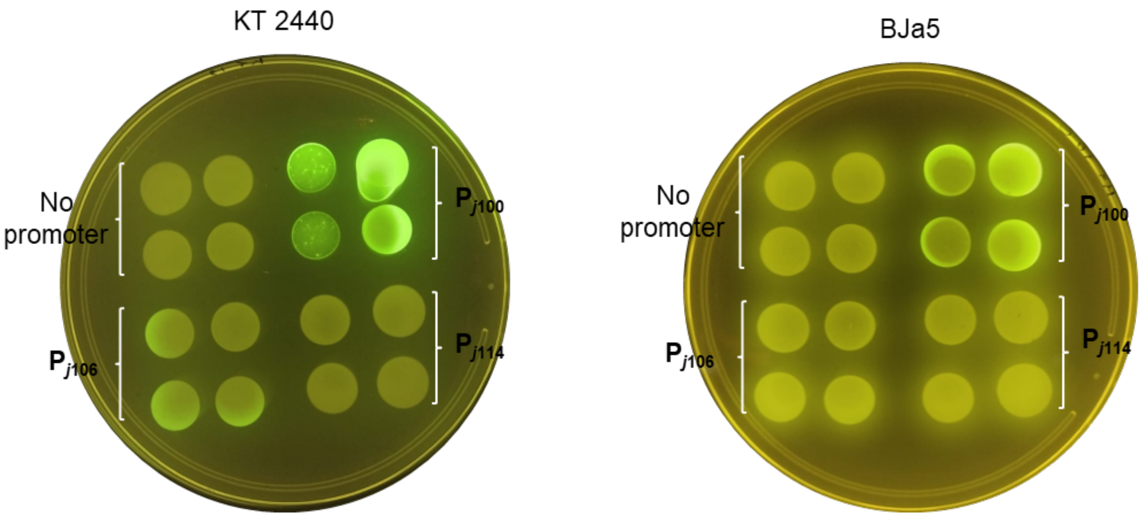

Figure S2. Activity of the canonical P*j100*, P*j106* and P*j114* promoter at 30°C. The functionality of the canonical promoters BBa_J23100 (P*j*100), BBa_J231006 (P*j*106) and BBa_J23114 (P*j*114) was evaluated at 30°C in *P. putida* KT2440 and in *Pseudomonas sp.* BJa5 using the GFP reporter gene and the empty vector pSEVA231. The plasmid containing GFP without promoter (No promoter) was used as a negative control. The assay was performed three times and similar results were obtained.

Table S1. Transformation efficiencies of plasmids in the BJa5 strain (UFC/µg DNA).

	Plasmid		
**Strain**	pSEVA228	pSEVA231	pSEVA247y
BJa5	10^8	10^8	10^7
